# Progress in Application of Silane Coupling Agent for Clay Modification to Flame Retardant Polymer

**DOI:** 10.3390/molecules29174143

**Published:** 2024-08-31

**Authors:** Yongwei Hu, Yong Liu, Shihao Zheng, Wendong Kang

**Affiliations:** 1 School of Resource & Environment and Safety Engineering, Hunan University of Science and Technology, Xiangtan 411201, China; hyongwei2022@163.com (Y.H.); z_henry2022@163.com (S.Z.); kangwd12@hnust.edu.cn (W.K.); 2 Hunan Engineering Research Center for Fire and Explosion Prevention Materials and Equipment in Underground Spaces, Xiangtan 411201, China; 3 Key Laboratory of Fire and Explosion Prevention and Emergency Technology in Hunan Province, Xiangtan 411201, China

**Keywords:** clay, flame retardant, modification, γ-aminopropyl triethoxysilane, γ-(2,3-epoxypropoxy)propytrimethoxysilane, intumescent flame retardant

## Abstract

Polymer composites are widely used in various fields of production and life, and the study of preparing environmentally friendly and flame retardant clay/polymer composites has gradually become a global research hotspot. But how to efficiently surface modify clay and apply it to the field of flame retardant polymers is still a potential challenge. One of the most commonly used surface modification methods is the modification of clay with silane coupling agents. The hydrolysable groups of the silane coupling agent first hydrolyze to generate hydroxyl groups. These hydroxyl groups then undergo a condensation reaction with the hydroxyl groups on the surface of the clay, allowing for organic functional groups to be grafted onto the clay surface. The organic functional groups and polymer matrix react to generate chemical bonds so that the composite material’s interface is more closely combined. Thus, the dispersion of clay in the organic polymer material and the compatibility of the two is better, which improves the flame retardant effect of the composite material. This paper introduces the classification of a silane coupling agent and the mechanism and process of silane coupling agent-modified clay, outlines the mechanism of silane coupling agent-modified clay flame retardant polymers, reviews the research results on flame retardant polymers of various clays after surface treatment with silane coupling agents in recent years, and highlights the synergistic flame retardant effect of clay and flame retardant organized by silane coupling agents. Finally, it is found that the current research in the field of silane coupling agent-modified clay in flame retardants is focused on the modification of montmorillonite, sepiolite, attapulgite, and kaolinite by KH-550, KH-560, and KH-570, and the development trends in this field are also prospected.

## 1. Introduction

With the development of the polymer discipline, polymer materials are now widely used in various fields, such as electric power, military and transportation. However, when they burn, they produce large amounts of toxic fumes that threaten people’s lives and health and affect the stable development of society and economy. Especially in densely populated places that cannot easily be evacuated, serious fires caused by polymer combustion often result in mass deaths, injuries and major property damage. For example, in 2022, the “11–21” special major fire accident in Anyang, Henan Province, China, was caused by the burning of a large number of knitted fabrics, raw cotton and plastic products [1]. In order to solve the problem of the high flammability of polymer materials, polymer/inorganic nano-filler composites with flame retardant properties have become a research hotspot in recent years. Numerous researchers have studied the flame retardant properties by functionalizing and modifying various inorganic nanofillers and adding them to polymer matrices [2,3,4,5,6,7,8,9].

**Figure 1:**
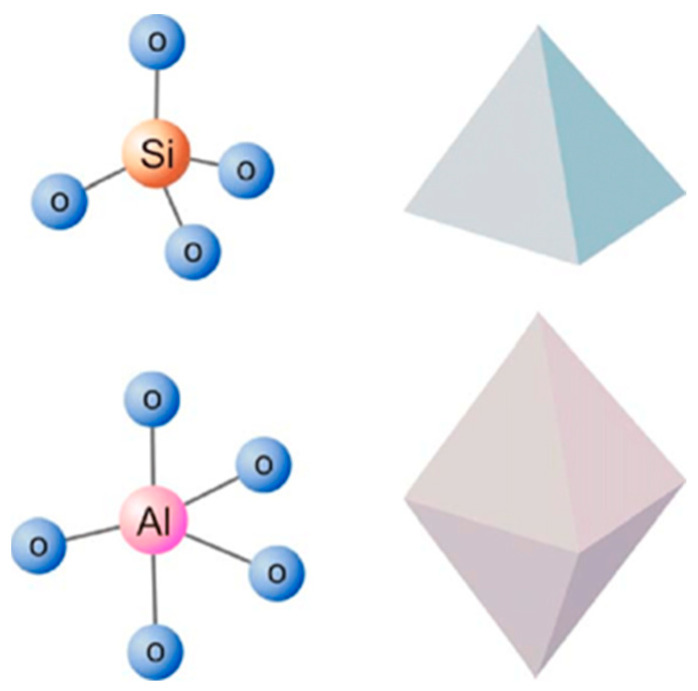
Tetrahedron and octahedron geometric forms [18].

**Figure 2:**
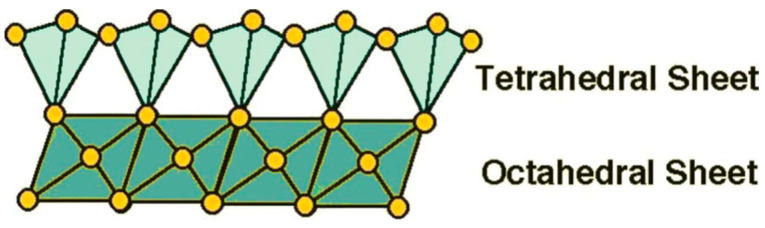
Structure of 1:1 layer silicate illustrating the connections between tetrahedral and octahedral sheets [18].

**Figure 3:**
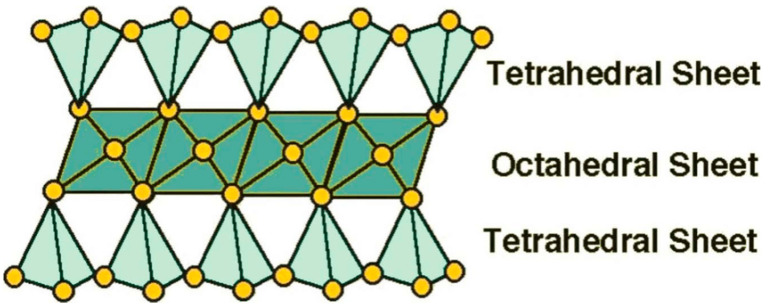
Structure of 2:1 layer silicate illustrating the connections between sheets [18].

Among all inorganic nanofillers, natural clay minerals have been incorporated into various polymer matrices to prepare nanocomposites with improved flame retardant and mechanical properties due to their unique structure, good thermal stability and low price [10]. Among the advantages mentioned above, the unique structure of clay is worth highlighting. The layered siloxane skeleton of the clay is constructed from individual [SiO_4_]^4−^ tetrahedra connected by sharing most of the angular tops and, thus, extending continuously and repeatedly in two dimensions [11]; therefore, it is also known as a tetrahedral sheet. All cations bound to the lamellar silica–oxygen skeleton are present in the octahedral voids formed by a layer of reactive oxygen and a layer of OH^−^, called octahedral sheets. The geometric forms of the tetrahedron and octahedron are shown in Figure 1. The tetrahedral sheets and octahedral sheets form the structural unit cell lamellae of clays [12,13]. Because of the different proportions of tetrahedral and octahedral sheets within the unit layer of clay minerals, they can be categorized into two main structural types, namely, the 1:1 type and the 2:1 type. In the 1:1-type clay structure, the apical oxygen of the tetrahedral sheet replaces one of the hydroxyl groups of the octahedral sheet, and the structure is shown in Figure 2. This type of clay includes kaolinite, serpentine, etc. In the 2:1 type of clay structure, two-thirds of the hydroxyl groups in the octahedral sheet between the two tetrahedral sheets are replaced by the apical oxygen of the tetrahedral sheet, and the structure is shown in Figure 3. This type of clay includes montmorillonite, sepiolite, mica, etc. [14,15,16]. However, clays, as a type of inorganic silicate mineral, are poorly compatible with organic polymer matrices, and research results point to the fact that clays, while strikingly affecting some of the fire resistance properties of polymers, are insufficient for commercial applications. Because they cannot act as stand-alone flame retardants in important fire tests [17]. Therefore, in order to avoid agglomeration and, thus, achieve uniform dispersion of inorganic nanophases in organic polymers, or to combine clays with conventional flame retardants to satisfy the fire test, the organic modification of clays has attracted much attention.

Silane coupling agent modification is an important method in the organic modification of clay. Silane coupling agent, as a modifier, has a development history of more than 70 years; the earliest application was in the field of polymer composites. This class of organ silanes was first used to improve the properties of glass fibers and also to enhance the properties of resin-based composites. In recent years, numerous scholars have reviewed the applications of silane coupling agents. In 2008, S. Shokoohi et al. [19] introduced the application of silane coupling agents in polymer matrix composites and described the coupling mechanism and bonding theory of the silane coupling agents. In 2010, Y. Xie et al. [20] summarized the advances in interfacial properties between natural fibers and polymers by silane coupling agents. In 2018, J. P. Matinlinna et al. [21] summarized the progress of silane coupling agents in dental applications. In 2020, F. Ahangaran et al. [22] investigated the progress of the modification of metal oxide nanoparticles by silane coupling agents in various applications. In 2021, T. Aziz et al. [23] summarized the progress of silane coupling agents in various applications.

For a long time, silane coupling agents have been used to improve the adhesion and compatibility between different materials, and their role in the performance and structure of polymer materials is particularly important. At present, there are more applications of amino silane coupling agent and modified amino silane coupling agent. With the progress of science and technology, an increasing number of silane coupling agent types are being developed. Commonly used silane coupling agents are epoxy silanes, vinyl silanes, long-chain alkyl silanes, multifunctional polymer silanes, and other newly developed types. Silane coupling agents can be used as inorganic fillers, such as glass fiber, kaolinite, volcanic ash, silica, and other surface treatment agents, improve the adhesion of inorganic fillers and polymers, so that the overall physical properties and durability can be further improved; as an adhesion promoter between difficult-to-bond materials such as polyolefins and special types of rubber; and it can also be used together with silicone emulsion as textile additives to improve the performance of textiles.

As modifiers of clay, surfactant modification is the insertion of organic ions and strong polar organic molecules into the interlayers of clays through reactions such as ion exchange or physical adsorption with clay [24]. This broadens the interlayer spacing of clay, thus obtaining excellent lipophilicity of clay. Silane coupling agents, on the other hand, act as a bridge between the clay and the polymer matrix. The silane coupling agent makes the clay lipophilic by condensing the reactive silica hydroxyl groups produced by silane hydrolysis with the hydroxyl groups on the surface and end face of the clay to form covalent bonds. This results in better compatibility between the two [25,26,27]. However, silane coupling agents as clay modifiers have some drawbacks. For example, some sulfur-containing silane coupling agents, when used in rubber, produce volatile organic compounds in industrial production [28]. Also, excessive addition of silane coupling agents to the polymer matrix can result in a loss of mechanical properties of the composite material [29]. Compared to silane coupling agents, surfactants are better able to expand the interlayer spacing of the clay, which facilitates the clay to reach the exfoliated state. Thus, making it easier for the polymer molecular chains to enter into the channels of the clay. However, scholars such as S.-L. Bee et al. [30] have argued that conventional surfactants tend to be thermally unstable, as they do not have an effective connection to the clay surface. This has become a significant limitation in industrial production processes such as plastic extrusion molding, especially in the production of polymers with high melting points such as polyetherimides. In contrast, when silane coupling agent-modified clay is used, covalent bonds form between the two, which are not interacting with the clay’s surface through simple electrostatic interaction or physical adsorption as surfactants. This improves the compatibility of the clay with the organic matrix while obtaining better organic phase stability than the surfactant-modified clay. Therefore, more scholars have focused on the modification of clays using organic silane. In order to improve the comprehensive utilization efficiency of clay and promote the application of silane coupling agent-modified clay in the field of flame retardants, in this paper, the research progress on different silane coupling agent-modified clays in the field of flame retardant polymer is reviewed, and future research directions on silane coupling agent-modified clays in the field of flame retardancy is also prospected.

## 2. Overview of Silane Coupling Agent-Modified Clay

A coupling agent is a kind of compound that can improve compatibility between inorganic materials and organic polymers, and there are many types of coupling agents, mainly silane coupling agent, titanate nitride coupling agent, aluminate coupling agent, bimetallic coupling agent, phosphate coupling agent, borate coupling agent, chromium complexes and other higher fatty acids, alcohols, esters of the coupling agent, etc. [31], of which silane coupling agent has the most wide range of use at present [32]. Silane coupling agent is a commonly used modifier for the organic modification of clays, and its structural formula is usually expressed as Y(CH_2_)_n_SiX_3_. Organic functional groups can be grafted onto the clay surface by silane coupling agents. The grafting of organic functional groups onto the clay surface enhances the interaction between the clay and the polymer matrix. This improved interaction not only enhances the dispersion of the clay within the polymer but also strengthens the composite material, thereby improving its flame retardant properties [33,34,35,36].

### 2.1. Classification of Silane Coupling Agents

Silane coupling agents can be classified according to the following different organic functional groups: vinyl-based silane coupling agents, chlorinated hydrocarbon silane coupling agents, ammonia hydrocarbon silane coupling agents, epoxy hydrocarbon silane coupling agents, methacryloyloxyalkyl coupling agents, sulfur-containing hydrocarbon silane coupling agents, pseudo halogen silane coupling agents, and quaternary ammonium hydro carbonyl silane coupling agents. The development of silane coupling agents with a better modification effect is an important research direction to expand the application range of silane coupling agents. Table 1 below lists the names, nicknames and chemical formulas of the currently used silane coupling agents.

### 2.2. Mechanism of Silane Coupling Agent-Modified Clay

Many theories have been proposed for the mechanism of silane coupling agents, such as chemical bonding theory, surface infiltration theory, deformation layer theory [37], reversible equilibrium theory [38], etc. Among them, the chemical bonding theory and surface infiltration theory have been recognized by more scholars, but these two theories still cannot explain all the silane coupling agent action mechanisms alone.

**Figure 4:**
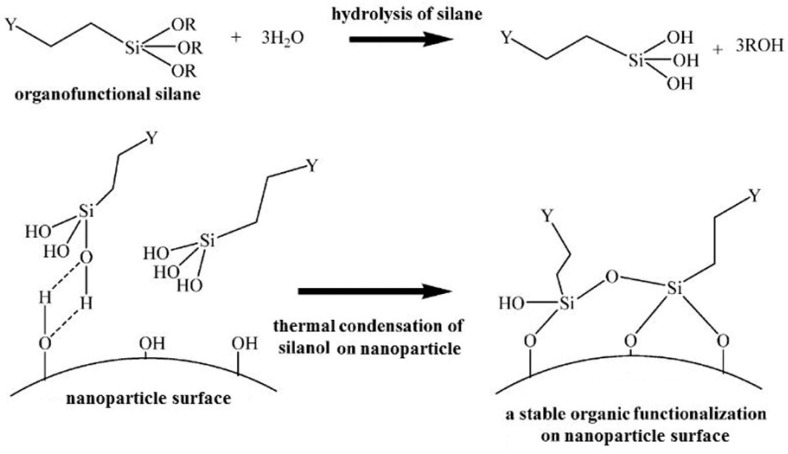
Schematic diagram of the silane coupling agent’s coupling process [22].

The influential theory of chemical bonding suggests that silane coupling agent has two different chemical functional groups [39]. B. Arkles [40] was the first to put forward the theory, and the coupling process of the silane coupling agent is summarized in four reaction steps (Figure 4). The three hydrolysis groups connected to silicon in the first step are hydrolyzed to silicone hydroxyl groups Si-OH. In the second step, Si-OH is dehydrated and condensed between them to form oligosiloxanes containing Si-OH. In the third step, Si-OH in the oligomers forms hydrogen bonds with hydroxyl groups on the surface of the substrate. And in the fourth step, covalent bonds are formed connecting them to the substrate in the process of curing accompanied by the dehydrating reaction during the heating process. Therefore, the silane coupling agent bridges the gap between the clay and the organic matrix. It can covalently graft various types of functional groups on the surface of the clay, thus changing the clay from hydrophilic and lipophobic to hydrophobic and lipophilic. This can effectively improve the dispersion of the clay in the organic matrix which, in turn, improves the flame retardant application of the clay in polymers. However, in practice, there is still a problem that the theory cannot explain. That is, the silane coupling agent can not react with some organic substrates or inorganic filler chemical bonding, but it can still improve the compatibility between the two to a certain extent.

The surface infiltration theory was first proposed by W. A. Zisman [41]. According to this theory, if excellent interfacial bond strength is to be achieved, the matrix resin should efficiently wet the inorganic filler. The silane coupling agent can rapidly wet the surface of inorganic materials because of its low surface tension, so that the polymer has strong physical adsorption at the interface between the organic and inorganic phases. However, the surface infiltration theory ignores the effect of the chemical bonding reaction between silane coupling agent and some organic matrix or inorganic filler on the compatibility of the two.

Therefore, combining chemical bonding theory with surface infiltration theory can provide a more complete description of the series of interactions that occur when silane coupling agents modify inorganic fillers.

When selecting a silane coupling agent, it is usually necessary to consider the chemical properties of the organic matrix and the organic functional groups contained in the silane coupling agents, which is an important condition for the composite materials to obtain the best performance. For example, for unsaturated polyester, vinyl silane coupling agents, epoxy silane coupling agents, and methacryloxy silane coupling agents are well compatible. For polyurethane, amino silane coupling agents are compatible. For epoxy resin, epoxy silane coupling agents or amino silane coupling agents are recommended. For phenolic resin, amino silane coupling agents and urea-based silane coupling agents are compatible. For olefin polymers, vinyl silane coupling agents are compatible. For sulphur vulcanized rubber is compatible with sulfur-containing alkyl silane coupling agents [19]. On the other hand, the modification effect of different silane coupling agents on clay is not exactly the same. This is due to the different grafting rate of each silane coupling agent on the clay surface and the molecular structure of the silane coupling agents. Tian et al. [42] compared the modification effects of KH-550 (containing amino group), KH-560 (containing epoxy group), and KH-570 (containing unsaturated double bond) on montmorillonite, and found that the grafting rate was 29%, 27%, and 13%, in that order. They also found that the montmorillonite layer spacing modified by KH-560 had a large increase of 0.3 nm, while the montmorillonite layer spacing modified by KH-550 and KH-570 had smaller increases of 0.12 nm and 0.08 nm, respectively. This is attributed to the difference in the number of silane molecules entering the montmorillonite interlayers. The epoxy bonds in the KH-560 molecules are easily hydrolyzed. After hydrolysis, the silicon oxygen bonds and epoxy bonds in the coupling agent molecules easily bond with the hydroxyl groups on the surface of the montmorillonite layer. Therefore, a large number of silane molecules enter into the montmorillonite interlayer. KH-570 contains double bonds that are not easy to hydrolyze, and fewer silane molecules are bonded to the hydroxyl groups on the surface of the montmorillonite, thus fewer silane molecules enter the interlayer of the montmorillonite. KH-550 also contains amino groups that are easy to hydrolyze. But after hydrolysis, only the silicon oxygen bonds are bonded to the hydroxyl groups on the surface of the montmorillonite. So, the silane molecules entering the interlayer of the montmorillonite are fewer than those of KH-560 but more than that of KH-570. In conclusion, when selecting silane coupling agent for surface modification of clay, it is necessary to consider not only whether beneficial chemical reactions can occur between the organic functional groups of silane coupling agents and the organic matrix but also whether the modification effect of silane coupling agents on a specific clay is excellent.

### 2.3. Silane Coupling Agent-Modified Clay Process

There are two main processes for the surface modification of clay using silane coupling agents, as follows: wet modification and dry modification.

Wet modification refers to the addition of well-dispersed silane coupling agents into the modification system of clay slurry prepared by a certain solid–liquid ratio with constant stirring, the modification is carried out at a certain temperature, and, finally, silane coupling agent-modified clay is obtained after filtering, washing, drying and other operations. Li et al. [43] added sepiolite to anhydrous ethanol and successively added an appropriate amount of KH-560, deionized water and KH-602 in a reflux mixing process. Then filtered, washed, dried, and milled the slurry, drying and grinding to produce KH-560 and KH-602 composite modified sepiolite.

Dry modification refers to a process in which clay and silane coupling agents are loaded into specific equipment for mixing, stirring and heating according to a certain material ratio. The dry modification process requires less production equipment, without dehydration and drying. The advantages of dry modification also include simple process, easy to control the operation, low production cost, and low pollution to the environment. This method has a wider scope of application, especially for non-water-soluble modifiers. Zheng et al. [44] added dried kaolinite and KH-570 into a high-speed mixer and stirred it, to produce organic modified kaolinite.

The advantages of the wet modification process are that there are many adjustable factors in the process, such as better dispersion of modifier, uniform surface coating, etc. Therefore, the modification efficiency of wet modification is higher, so it is commonly used for laboratory research. However, compared with dry modification, the wet modification process requires pre-slurry configuration, postfiltering, drying, and dewatering, which makes the process complicated and the modification cost higher. And some scholars [45] modified attapulgite (attapulgite is a crystalline hydrated magnesium aluminum silicate with a unique three-dimensional structure and has a fibrous morphology) by different modification methods. They found that the mechanical force can effectively disperse attapulgite and provide more energy to enhance the coupling effect between silane coupling agents and attapulgite during ball milling modification. Therefore, for industrial production, dry modification is usually chosen. This has led to the fact that the wet process is relatively mature, and there is a wealth of research on the related modification mechanisms. However, the dry process has been poorly explored. In order to make silane coupling agent-modified clay more widely and efficiently used in the industrial production of flame retardant composites, researchers are recommended to do more work on dry modification, such as the mechanism of dry modification and the factors affecting the effect of dry modification.

## 3. Silane Coupling Agent-Modified Clay Flame Retardant Polymer

Based on the above advantages of silane coupling agents, numerous scholars have applied silane coupling agents in specific experiments on polymer/nano-clay. They have characterized the flame retardant properties of the composites and discussed the flame retardant mechanisms. J. R. Beryl et al. [46] detailed the synthesis techniques of nano-clay-based composites in their review. In this section, the mechanism of silane coupling agent-modified clay flame retardant polymers is summarized. Several specific examples of nano-clay applications to flame retardant polymers are reviewed and discussed.

### 3.1. Mechanism of Silane Coupling Agent-Modified Clay Flame Retardant Polymer

**Figure 5:**
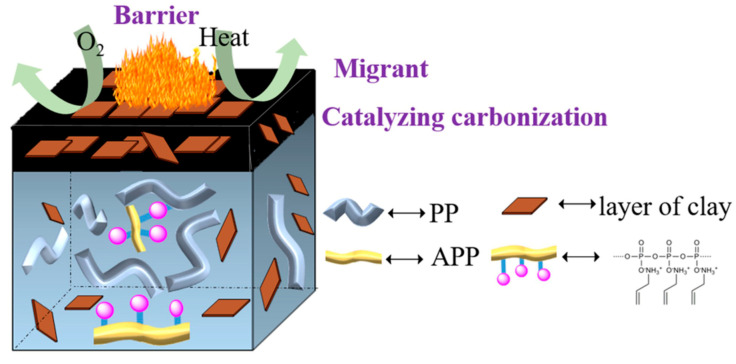
Schematic diagram of the char formation and barrier mechanisms [47].

The key to clay’s improvement of the flame retardancy of polymers is the formation of a char layer during the combustion process of the composite materials. Clay modified by silane coupling agents can uniformly distribute in the polymer matrix. In the process of combustion, the clay will generate a uniform dense carbonaceous silicate layer. The carbonaceous silicate layer can block the heat generated by the combustion of the materials and the outside air to the internal penetration of the materials. Thereby slowing down the supply of heat and oxygen for combustion inside the materials. The carbonaceous material can also play a role in inhibiting the volatilization and spillage of flammable substances produced during the combustion process. Thereby enhancing the flame retardant properties of the material. The mechanism of silane coupling agent-modified clay flame retardant polymers can be summarized in the following three points:

(1) Char formation and barrier mechanism: The formation of a char layer during polymer cracking is a very complex process. It contains several steps, such as the generation of conjugated double bonds, cyclization, aromatization, melting of aromatic rings, formation of turbulent carbon, and graphitization. Since most clays contain Lewis acids, they have a catalytic role in char formation, and their char formation chemistry proceeds as follows: Firstly, the thermal decomposition of clays and polymers by heat transfer from an external heat source or flame to the material leads to the formation of certain proton catalytic centers in the interlayers of the silicates, which are concentrated on the surface of the combusted material. Subsequently, the polymer reacts with it and the conjugated polyene produced by the reaction is then aromatized, cross-linked, and catalytically dehydrogenated to form a surface char layer. Finally, the char layer is inserted into the silicate layer to become the char-containing combustion residue. When the clay is used in silane coupling agent surface modification, it can be uniformly dispersed on the nanometer scale. Therefore, these layers of clay can not only limit the movement of the macromolecular chains but also isolate the energy and oxygen exchange. At the same time, the clay uniformly dispersed in the polymer is pushed to the surface to be enriched during the combustion of the materials due to the outward spillage of the volatile gases generated. This increases the viscosity of the combustion of the molten materials to promote the formation of a continuous network of char layers, thus having high flame retardant efficiency [47]. The mechanism is shown in Figure 5.

(2) Free radical capture mechanism: Clay modified by silane coupling agents can uniformly distribute in the polymer matrix. The small amount of transition metal ions (e.g., Fe^3+^ and Ni^3+^) contained between the clay layers can efficiently capture free radicals during the combustion process of composite materials and improve the flame retardant performance of the materials [48].

(3) Flame retardant mechanism of crosslinked silane molecules: Crosslinked silane molecules and clay can produce silicon-carbon synergistic flame retardant effect. Usually, silane molecules are first hydrolyzed in water to form Si-OH, and react with clay and their own hydroxyl groups through condensation reaction, thus forming a cross-linked structure. During the combustion process, silane is thermally decomposed to form a dense protective layer of silica nanoparticles on the clay layer and promote the formation of a dense carbon layer on the clay, resulting in a synergistic flame retardant performance [49]. It not only effectively prevents the escape of flammable gases, but also enhances the effect of blocking oxygen and heat.

### 3.2. Silane Coupling Agent-Modified Montmorillonite

**Figure 6:**
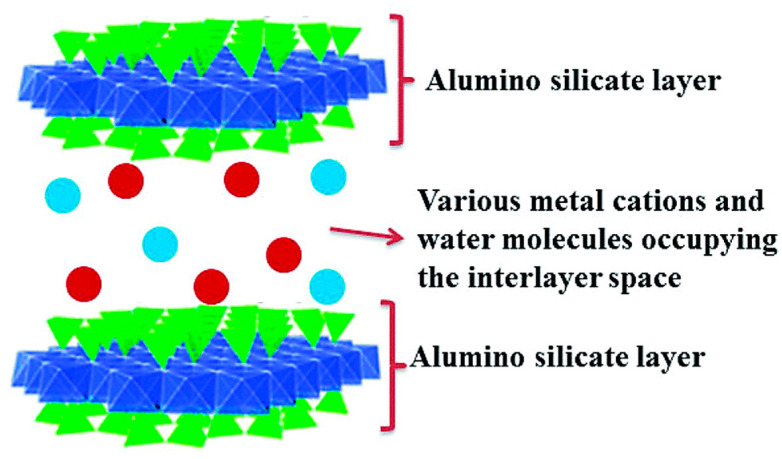
Schematic diagram of montmorillonite structure [52].

Montmorillonite (MMT) is one of the most typical clays with the theoretical structural formula of (Na,Ca)_0.3_(Al,Mg)_2_Si_4_O_10_(OH)_2_·nH_2_O. Its structure is shown in Figure 6. Due to its halogen-free and nontoxic properties, low price, and huge resource reserves, it has attracted the attention of many scholars and is widely used in the field of flame retardant [50,51].

**Figure 7:**
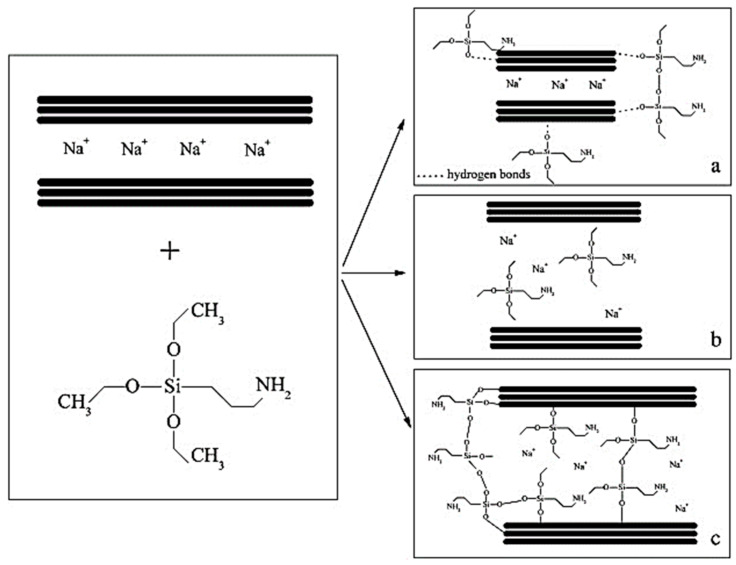
Three interactions of silane coupling agent with MMT (γ-APS as an example): (**a**) adsorbed silane; (**b**) intercalated silane and (**c**) grafted silane [53].

P. T. Bertuol [53], using KH-550, investigated the interaction between silane coupling agents and MMT, as shown in Figure 7. The modification of MMT by the silane coupling agent resulted in an increase in the basal spacing from 12.1 Å to 20.5 Å. This is due to the fact that the silane molecules grafted or inserted into the interlayer space make the attraction between the MMT layers less attractive, thus increasing the layer spacing. This enables MMT to be better dispersed in the polymer matrix, which can increase the density of combustion residual carbon and give full play to the flame retardant effect of the condensed phase. Sun [54] compared the flame retardancy of composites coating without MMT, composites coating with untreated MMT and composites coating with silane coupling agent KH-560-modified MMT. The latter showed a reduction in the heat release rate, mass loss, and mass loss rate, and a significant enhancement of the flame retardancy compared to the former two. This is due to the fact that part of the polymer enters the pores of the MMT during the mixing process, between the coating solution and the nano-MMT. Penetration and bonding of the resin drives the MMT flakes to open up, peel off, and disperse in the polymer matrix, resulting in an MMT nanocomposite coating. Uniformly dispersed MMT promotes the formation of a barrier layer on the burning surface. Li et al. [55] modified MMT using KH-550 and incorporated it into epoxy resin. It was found that the glass transition temperature of the epoxy resin with the addition of modified MMT increased from 156 °C to 165 °C, and the heat resistance was enhanced. This is due to the increase in cross-linking density after the composite of silanized MMT and epoxy resin, which hinders the movement of molecular chains. In addition, the silane coupling agent-modified MMT itself has high thermal stability. Therefore, the heat resistance of the composites was improved. Yu et al. [56] prepared phenolic resin/organic MMT nanocomposites from MMT modified with silane coupling agent KH-560 (hydrolysis and nonhydrolysis, respectively). After comparison, they found that KH-560 entered the MMT interlayer more under nonhydrolyzed conditions. This led to a uniform dispersion of MMT in the resin, making the MMT lamellae more effective in blocking oxygen and heat. Therefore, optimization research on modification conditions is a feasible way to improve the modification effect of silane coupling agent.

Compared with the modification of MMT by a single modifier, the organic MMT obtained by composite modification with a suitable silane coupling agent and other modifiers tends to give the composites better flame retardant properties. The ionic surfactant and silane coupling agent composite-modified MMT can be more uniformly dispersed in the matrix. Composite modifications involving metals and their compounds often provide MMT with flame retardant and smoke-suppressant abilities. Wang et al. [57] prepared three composites modified MMT (K550-C-MMT, K560-C-MMT, K602-C-MMT) based on quaternary ammonium modified MMT (C-MMT) respectively with KH-550, KH-560, and KH-602, and aramid-based epoxy resin/MMT composites. The thermal stabilities of the composites were K602-C-MMT, K550-C-MMT, K560-C-MMT, C-MMT in descending order. The temperature of a 5% and 50% mass loss of the composites prepared with K602-C-MMT was enhanced by 8.36 °C and 7.50 °C, respectively, compared with that of pure EP. This is due to the fact that the composite-modified MMT is exfoliated from the lamellae in the epoxy resin matrix and is able to be more uniformly dispersed in the resin matrix. This results in a stronger interaction with the resin and the thermal stability of the composite is improved. Yang et al. [58] compared the flame retardant properties of silane coupling agent-modified MMT and composite modified MMT with copper sulfate and silane coupling agent filled in poly(vinyl chloride) (PVC) resins prepared from composites. It was found that the composite-modified MMT could promote the early cross-linking of the PVC chain, increase residual carbon content, and reduce the smoke by 45–50%. The generation of benzene and its derivatives during the combustion of PVC is the main factor in the formation of smoke. The metal and its compounds are added to the composites through the surface modification of MMT. When the composites are thermally degraded, they react with the hydrogen chloride from off the PVC to generate metal chlorides, which catalyze the alkylation reaction, as well as dehydrochlorination, leading to the formation of trans-polyenes and, at the same time, cross-linking occurs to induce the formation of char. This catalyzes the alkylation reaction and also catalyzes the dehydrochlorination, leading to the formation of trans polyenes instead of cyclizing to benzene and its derivatives. Cross-linking also occurs to induce coke formation. All of these effectively improve the flame retardant and smoke inhibition performance of the composite materials. When composite modification is used, the proportions of different modifiers are recommended to be studied in depth in order to obtain the optimal modification effect.

MMT can also produce excellent synergistic flame retardant effects with other flame retardants after treatment with silane coupling agents. Zhou et al. [59] prepared microencapsulated red phosphorus, phosphorus-based flame retardant RC200, and modified MMT flame retardant acrylonitrile-butadiene-styrene copolymer (ABS) resins. The LOI value of the composites increased from 18.3% to 24.9% and the UL-94 rating up to V-0. The formation of dense and solid residual char is the key to the effectiveness of phosphorus flame retardants for ABS. During pyrolysis and combustion, silanized MMT contributes to the forming of a dense char layer. This confirms a significant flame retardant synergistic effect between the silane coupling agent-modified MMT and microencapsulated red phosphorus and RC200 systems. Xu et al. [60] prepared organic MMT/nano-Sb_2_O_3_/BEO/PP composites by melting a mixture of brominated epoxy resin (BEO), polypropylene (PP), silanized MMT, and nano-Sb_2_O_3_ particles. The experiment proved that the 11.6% increase in the LOI of PP-based composites over pure PP and the V-0 rating of UL-94 were due to the addition of silanized MMT. These enhancements may be due to two reactions, i.e., the gas-phase reaction of Br-Sb and the condensed-phase reaction of the silane coupling agent-modified MMT. Therefore, there is good synergy between the silane coupling agent-modified MMT and Br-Sb. Research on the synergistic flame retardant effect of silane coupling agent-modified MMT with other flame retardants is a good area for research.

Both the octahedral and tetrahedral layers of MMT have a considerable degree of ionic substitutions. These substitutions lead to negative charges in the structural unit layers. These negative charges are balanced by exchangeable cations between the layers of the structural units and at the edges of the unit layers, resulting in a high cation-exchange capacity of MMT. Therefore, when many scholars carry out surface modification of MMT, surfactants such as quaternary ammonium salts are usually used for organic modification. However, MMT has a small number of silanol groups at the edge of its particles which graft silane coupling agent. Thus, the compound modification of MMT with ionic surfactant and silane coupling agent is more conducive to its dispersion in the matrix [61], so as to better perform its flame retardant effect.

### 3.3. Silane Coupling Agent-Modified Sepiolite

**Figure 8:**
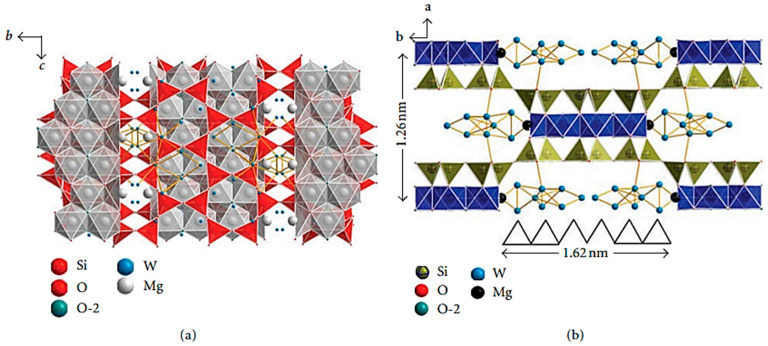
Crystal structure of sepiolite ((**a**) along a-axis direction; (**b**) along c-axis direction) [64].

Sepiolite (SEP) is a 2:1-type clay with an ideal structural formula of Si_12_O_30_Mg_8_(OH)_4_(H_2_O)_4_·8H_2_O [62], which is inexpensive and has abundant proven reserves [63]. Figure 8 shows the crystal structure of SEP along different axes. SEP contains nonhalogenated flame retardant elements, such as magnesium and silicon, which gives it a certain role in the flame retardant performance. And SEP has a fiber-like structure, which can inhibit the diffusion of oxygen and reduce the volatilization of thermal decomposition. At the same time, SEP can form a protective layer to block heat transfer. In addition, SEP also has the characteristics of small thermal conductivity and high temperature resistance, which give SEP a certain flame retardant effect.

**Figure 9:**
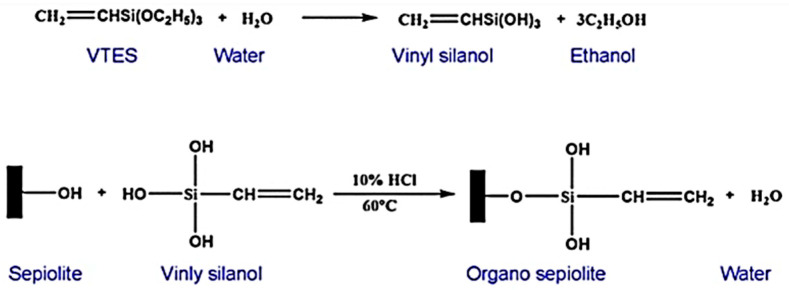
Schematic diagram of the mechanism of silane coupling agent-modified sepiolite [68].

Silane coupling agent-modified SEP can be uniformly distributed in the polymer matrix, effectively limiting the migration of the polymer chain. In addition, SEP, as a quality transmission barrier, can block or inhibit the transport of flammable vapors during combustion, thus improving the flame retardancy of the polymer materials. Figure 9 shows a schematic diagram of the mechanism of the silane coupling agent-modified SEP (with the silane coupling agent VTES, for example). Wang et al. [65] used the silane coupling agent KH-570-modified SEP and mixed with EP to prepare composite materials and then compared them and found that the addition of modified SEP effectively improved the flame retardant properties of the composites. When the contents of the modified SEP were 1%, 3%, and 7%, the corresponding PHRRs were 1066 kW/m^2^, 941 kW/m^2^, and 876 kW/m^2^, respectively. Compared with the PHRR of pure EP, the PHRRs decreased by 14%, 24%, and 29%, respectively. The sustained combustion time of the composites increased from 374 s to 392 s, 417 s, and 444 s. The LOI values of the composites increased from 21.1% to 21.4%, 21.8%, and 22.0%, respectively. During combustion, the modified SEP can form a stable carbon layer on the polymer surface. The reticular structure of the carbon layer can restrict the movement of polymer molecular chains. In addition, the blanket effect formed by the modification can reduce the rate of oxygen entering the polymer and diffusing between the polymers. H. C. Bidsorkhi et al. [66] prepared flame retardant ethylene vinyl acetate (EVA) composites using unmodified SEP and silane coupling agent KH-540 (containing amino)-modified SEP as materials, respectively. The incorporation of 5 wt% of unmodified and modified SEP in pure EVA increased the LOI values from 18.2% to 24.3% and 26.5%, respectively, and resulted in better mechanical properties and thermal stability. The improved flame retardancy is attributed to the insertion of SEP fibers into the EVA matrix, which limits the mobility of the EVA chain. In addition, SEP acts as a mass transport barrier, retarding or inhibiting the transport of flammable vapors during combustion.

The selection of a suitable silane coupling agent and other modifiers for SEP composite modification usually results in composites with better flame retardant properties. Yan et al. [67] used silane coupling agent KH-570-modified aluminum-phosphate-coated SEP for flame retarding epoxy resin. Adding 20% aluminum-phosphate-coated SEP to the epoxy resins, the LOI increased from 21.8% to 30.1% and the composite reached UL-94 V-0 grade. Aluminum phosphate, as a metal compound precipitated on the surface of SEP, gives SEP flame retardant and smoke suppressant properties. And the silane coupling agent improves the compatibility of SEP with the substrate and uniformly disperses the it. The composite modification of SEP using silane coupling agents and other modifiers is currently an important approach to SEP modification.

Silane coupling agent-modified SEP is used in combination with other flame retardants or inorganic fillers, which not only reduces the amount of additives of other flame retardants but also achieves a better flame retardant effect. Wang et al. [69] incorporated silane coupling agent KH-550-modified SEP fibers and nano-SiO_2_ into epoxy resin. The LOI of the pure epoxy resin was 21.8%, 28.7% after the addition of SEP alone, and 29.2% after the addition of nano-SiO_2_ alone. The LOI was higher than the previous three cases when SEP and nano-SiO_2_ were added together. And the LOI was the largest when the mass ratio of SEP/nano-SiO_2_ was 7:3, which was up to 30.3%. The silane coupling agent-modified SEP and other inorganic fillers are added to the polymer in a certain ratio, improving the compatibility of the inorganic fillers with the matrix, and the different shapes of the inorganic fillers penetrate the matrix to form a dense structure. The dense structure can effectively block the exchange of oxygen and heat and improve the flame retardant property of the composite materials. This is the reason why silane coupling agent-modified SEP produces a better flame retardant effect when compounded with other inorganic fillers. Liu et al. [70] compounded KH-550-modified SEP and conventional flame retardants to flame retard polyamide 6. It was found that the compounding of organic SEP, decabromodiphenyl ether, and Sb_2_O_3_ could greatly improve the flame retardancy and thermal stability of polyamide 6. And the addition of polytetrafluoroethylene, which has the effect of an anti-melting droplet, could further enhance the flame retardancy of the system. The LOI value increased from 21% to 32% and UL-94 to V-0 rating. The metal–organic skeleton of ZIF-8-grafted SEP (ZIF-8@SEP), through a silane coupling agent, can have a synergistic flame retardant effect with aluminum hypophosphite (AHP). ZIF-8@SEP promotes the formation of a denser carbon layer to shield against heat and oxygen transfer, and it adsorbs part of the smoke generated by AHP to reduce the release of composites via smoke. Li et al. [71] grafted SEP with ZIF-8 via the silane coupling agent EPTMS and added the product mixed with AHP to thermoplastic polyurethane (TPU) to study its flame retardant properties. In the TPU/8.0AHP/1.0ZIF-8@SEP composite system, the LOI reached 27.5%, and the fire rating reached UL-94 V-0. Research on the synergistic flame retardant effect of silane coupling agent-modified SEP with other flame retardants and the optimal additive amounts of both is a current research hotspot.

SEP has a large amount of reactive -OH because of the discontinuous structural unit layer, which is favorable for silane coupling agent grafting. Dissolving the cations in the octahedral position of SEP with acid also produces fresher -OH, which is more convenient for grafting. SEP also has a certain amount of exchangeable cations in its channels, and its cation exchange capacity is smaller than that of MMT but larger than that of kaolinite. But, unlike MMT, the lamellae on SEP are connected by weak covalent bonds, resulting in a limited degree of change in the lamellar spacing after the exchange of organic cations [72]. In summary, the surface modification of SEP is recommended to be conducted by grafting silane coupling agent. In addition, the SEP structure contains zeolitic water and coordination water filled inside the channels. In combustion, these water molecules are removed at about 100 °C and 350 °C, respectively. The resulting water vapor reduces the matrix combustion surface temperature and also dilutes the flammable gas concentration.

### 3.4. Silane Coupling Agent-Modified Attapulgite

**Figure 10:**
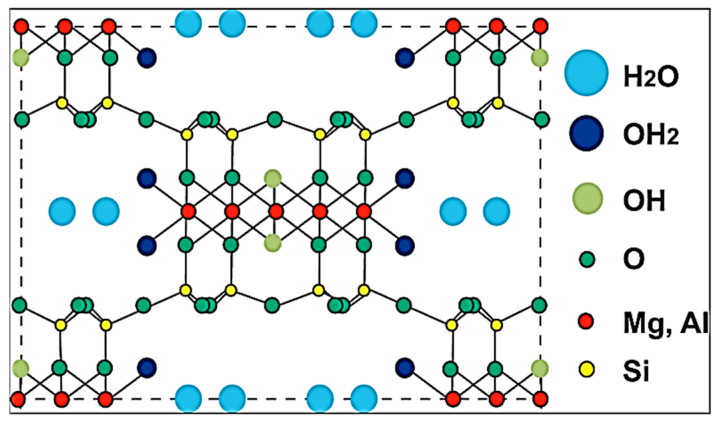
Atom arrangement in the projection plane of ATP [75].

Attapulgite(ATP), also known as palygorskite, is an aqueous magnesium–aluminum-rich silicate mineral between the chain structure and the layered structure, with a characteristic layered chain molecular structure, which is shown in Figure 10. Mineralogically, ATP belongs to the sepiolite family. Because of the special rod crystal structure, large surface area, pore structure, and other characteristics, it is widely used in chemical, metallurgy, food, paper, medicine, feed, construction, and other fields, with ATP having the reputation of being “universal soil”, “king of thousands of soil”, etc. [73,74].

The silane coupling agent can form covalent bonds with Si-OH groups and graft them onto the ATP surface. Wang et al. [76] first modified ATP nanorods with APTES (containing amino groups) and then mixed them with epoxy resin to prepare nanocomposites. They argue that only one ethoxyl group on the coupling agent was involved in the reaction. Jesionowski et al. [77] reported the mechanism of APTES molecules when interacting with ATP surfaces during the silicification process, with two of the ethoxyl groups on the coupling agent involved in the bonding reaction. Zhang et al. [78] prepared APTES-modified ATP as a novel addition in the preparation of ultrafiltration membranes. In their study, they noted that all three ethoxylates on the coupling agent were involved in the bonding reaction. Of the three grafted states, ATP is relatively more stable in the double-grafted state.

**Figure 11:**
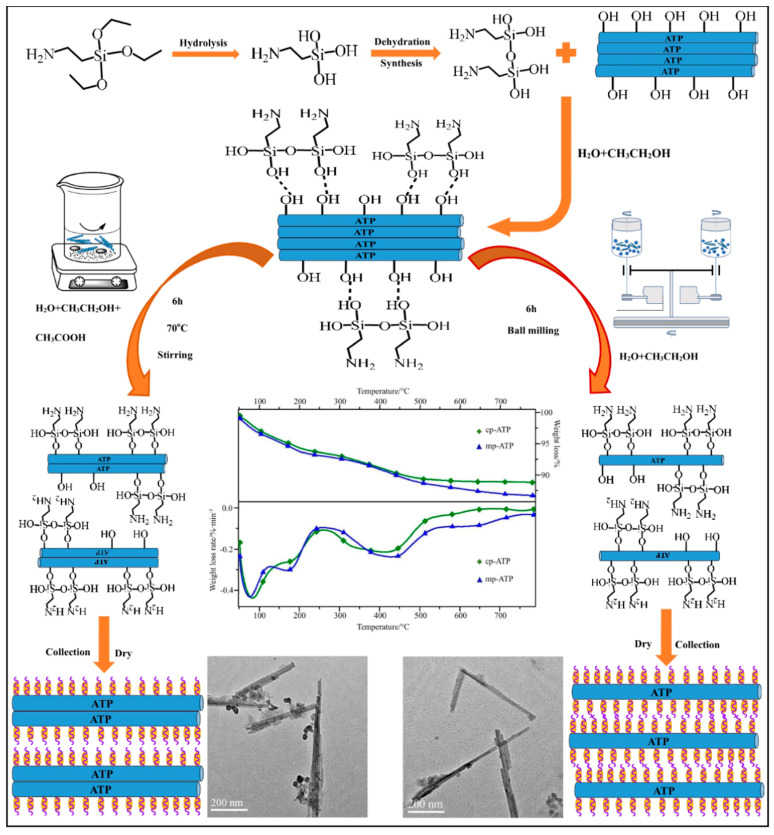
Schematic diagram of ATP modification by silane coupling agent (wet modification shown on the left and dry modification on the right) with TG, DTG curves, and TEM images of the ATP samples [45].

After surface treatment with silane coupling agent, ATP has better dispersion in the matrix and more exposed pores on the surface. This enables ATP to adsorb more smoke during the early stage of combustion of the composite material and decompose thermally to release water to cool down the combustion zone. And the vapor produced by decomposition also dilutes the concentration of combustible gases. During sustained combustion of the composite, the modified ATP also decomposes into more stable oxides, which form a dense segregation layer on the polymer surface. The segregation layer exerts the flame retardant effect in the condensed phase. Figure 11 shows a schematic diagram of the chemical modification, ball milling modification, and dispersion mechanism of ATP. Zhang et al. [79] used A-171 (containing unsaturated double bonds) to modify ATP and then added cross-linked polyethylene to prepare composite materials. Through comparison, it was found that the incorporation of modified ATP significantly reduced the internal voids of the low-density polyethylene materials and made the materials denser internally. Thus, the addition of modified ATP increased the decomposition temperature of the composite materials and the amount of combusted residual carbon increased. Bao et al. [80] used ball milling, activation, and KH-570 treatment to modify ATP sequentially. And then they modified cotton fabrics by sulfhydryl grafting. Finally, they grafted ATP onto the surface of cotton fabrics by a sulfhydryl-alkene click reaction. The resulting cotton fabrics showed excellent flame retardant properties with an LOI value increased from 18.5% to 25.6%. The reason is that in the heating process, ATP is decomposed into more stable oxides, such as SiO_2_, MgO, Al_2_O_3_, and other oxides. These oxides in the fiber’s surface form a dense isolation layer, thus blocking the penetration of oxygen, cutting off the heat transfer and inhibiting the degradation of cotton fabrics and the release of flammable volatile organic compounds. Gao et al. [74] prepared polyacrylic acid/attapulgite (PAA/ATP) nanocomposites by modifying ATP with KH-570. And they loaded the nanocomposites onto cotton fabrics, which made the nanocomplexes more durable. The nano-complexes were loaded onto cotton fabrics, which resulted in improved thermal stability and flame retardancy. The cotton fabrics treated with the composites had more residual charcoal, and the LOI value increased to 22.7% compared with the control fabrics. This is because ATP can be evenly dispersed on the surface of cotton fabrics. During combustion, ATP can absorb heat, decompose, and release water to cool the combustion zone. Gaseous water can dilute the concentration of combustible gases. Activated SiO_2_ and MgO(Al_2_O_3_) layers can form a dense barrier layer on the fiber’s surface. The barrier layer provides thermal insulation and smoke suppression, and it blocks oxygen penetration. This cuts off the heat transfer and inhibits degradation and the release of flammable VOCs in the gas phase. Zhang et al. [81] used KH-172 (containing unsaturated double bonds) to modify palygorskite and prepared ABS/palygorskite composites by melt blending method. It was demonstrated that silane coupling agent-modified palygorskite could increase the carbon formation of ABS resin and improve the thermal stability of ABS resin.

In addition, there is a synergistic flame retardant effect between silane coupling agent-modified ATP and intumescent flame retardant. Chen et al. [82] used KH-550 modified attapulgite (OATP) with a combination of ammonium phosphate and poly (1,3-propylenediamine-1,3,5-triazine-0-bicyclopentaerythritol phosphate) synthesized as intumescent flame retardant (IFR) for continuous glass fiber reinforced polyethylene (GFPE) composites synergistically. The addition of 4 wt% OATP to GFPE/IFR resulted in an LOI up to 31.3% and a significant reduction in the flame propagation rate. Zhou et al. [83] compounded WD-10 (containing long-chain alkyl)-modified palygorskite with piperazine–ammonium polyphosphate (PA-APP) into polypropylene. In the PP/PA-APP system, replacing the same mass fraction of PA-APP with 2 wt% modified palygorskite increased the LOI of the composite from 33.7% to 36.7%, and it achieved a V-0 grade in a vertical combustion test. The tensile strength and flexural strength increased by 10.9% and 13.6%, respectively, proving a significant synergistic effect between organically modified palygorskite and IFR in PP. These results are attributed to the fact that the metal oxides formed during the thermal decomposition of silane coupling agent-modified ATP makes the char layer more stable when subjected to heat. Thus, ATP slows down the degradation of IFR and generating a more uniform and dense char layer. The synergistic flame retardant effect of silane coupling agent-modified ATP with other flame retardants and their combination ratio is a hotspot in research.

ATP is a natural one-dimensional fibrous nanoclay with a structure similar to SEP, and the inside of the channels are filled with zeolitic water, coordination water, and constitution water. After surface treatment with silane coupling agent, the silanized ATP uniformly distributed in the polymer matrix can absorb heat and decompose to release water to cool the combustion zone. The vapor dilutes the concentration of combustible gases. The decomposition of the formation of reactive SiO_2_ and MgO (Al_2_O_3_) layer on the surface of the materials to form a dense isolation layer, which prevents the penetration of oxygen. The isolation layer can also cut off the heat transfer and inhibit degradation, and improves the flame retardant properties of the composite material.

### 3.5. Silane Coupling Agent-Modified Kaolinite

**Figure 12:**
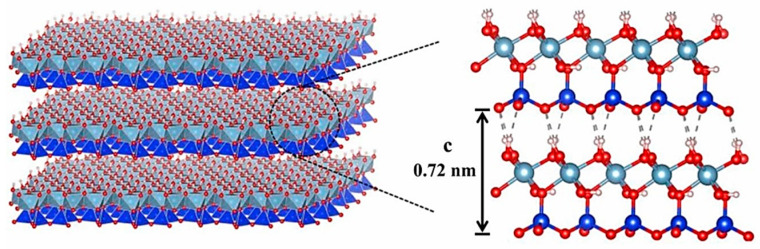
Schematic diagram of kaolinite crystals [86].

Kaolinite (Kaol) is a clay mineral with a great reserve [84], and its theoretical chemical formula is usually expressed as 2SiO_2_·Al_2_O_3_·2H_2_O. It has a typical crystal structure of 1:1-type clay, as shown in Figure 12. Because of its plasticity, bonding, dispersion, adsorption and strong chemical stability, it is widely used in industrial fields such as papermaking, ceramics, rubber and plastic, refractory materials, chemical industry, and building materials [85].

**Figure 13:**
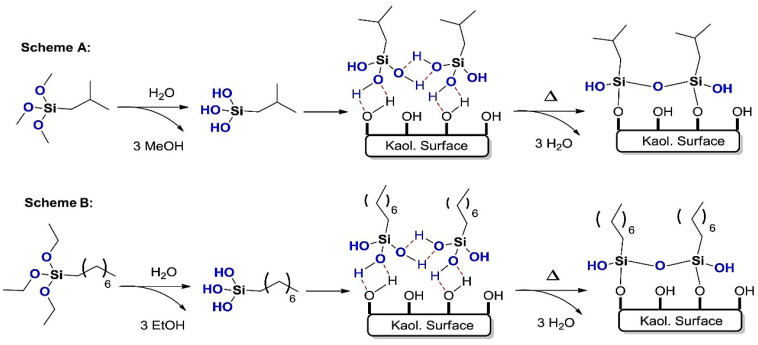
Schematic diagram of the mechanism of modification of Kaol by silane coupling agent (**A**: IBTMS and **B**: OCTEO as examples) [89].

Surface modification of Kaol by silane coupling agents results in its uniform dispersion in the polymer, thus improving the flame retardant properties of the composite. Figure 13 shows a schematic diagram of the mechanism of silane coupling agent-modified Kaol (using n-octyltriethoxysilane (OCTEO) and isobutyltrimethoxysilane (IBTMS) as examples). Zhang et al. [87] compared composites prepared with KH-792 (containing double amino)- and KH-550-modified ultrafine Kaol inserted into starch–chitosan (SCS) to prepare SCS-Kaol composites. It was found that the modified ultrafine Kaol formed a dense intercalation structure with SCS. The KH-792-modified ultrafine Kaol inhibited the crystallization of the polymers and dramatically enhanced the thermal stability of the polymers. The KH-550-modified ultrafine Kaol was favored the viscoelasticity of the composites. While improving the flame retardancy of the composites by modifying Kaol with a silane coupling agent, it is also recommended to consider other properties of composites such as mechanical properties, water and corrosion resistance, processability, and degradability.

Composite modification of Kaol with silane coupling agents and other substances usually results in composites with better flame retardant and smoke suppressant properties. Fan et al. [88] successively added SiO_2_, TiO_2_, and APTES to Kaol and incorporated the resulting products into epoxy resin. This improved the flame retardant and smoke suppressant properties and aging resistance of the epoxy resin. Compared with pure EP, the LOI value increased from 25.3 to 27.3, and the heat release rate, total smoke release, and smoke density were reduced by 36.9%, 14.3%, and 19.9%, respectively. The effect of silane coupling Agent-modified clays on epoxy resins has been summarized in a table as Supplementary Materials. The silane coupling agent-modified Kaol promoted the formation of a dense char layer during combustion, significantly preventing the heat and combustible gases from conducting to the outside. The nano-TiO_2_ adsorbed the gases generated during combustion, improved the smoke suppression of the composites, and catalyzed the oxidation of CO into CO_2_, while the SiO_2_ and TiO_2_ together improved the aging resistance of the polymers.

Silane coupling agent-modified Kaol compounded with other inorganic fillers or flame retardants usually has a better flame retardant effect. Zeng et al. [90] compounded KH-570-modified Kaol with flame retardant bisphenol A bis (diphenyl phosphate) (BDP) to flame retardant polyphenylene ether/high-impact polystyrene alloy. The LOI of composite material reached 32.2%. This is due to the fact that in addition to the flame retardant mechanisms of Kaol and BDP, there is also a silicon phosphorus complex mechanism between the two. At high temperatures, phosphorus catalyzes the formation of charcoal, while silicon increases the thermal stability of the charcoal layer, thus exerting the synergistic flame retardant effect of phosphorus and silicon. Zhang et al. [91] modified Kaol with KH-550 and Si-69 and filled it with carbon black in styrene butadiene rubber to study its flame retardant properties. The modified Kaol and carbon black cooperated with the filling to increase the temperature for the maximum thermal decomposition rate of the composite materials. The hybridization of spherical carbon black and platelet kaolinite will mutually promote the dispersion of the filler particles, strengthen the barrier effect of the filler on heat conduction, and improve the thermal stability of composites.

The octahedral and tetrahedral layers of Kaol are almost devoid of ion substitution. Thus, the structural unit layers have very little charge, low ion exchange capacity and adsorption capacity, and the spacing of the layers is small. The bonding between the layers is relatively strong, which leads to Kaol being difficult to interlayer. So, the surface modification of Kaol by silane coupling agent is an important means of its organic modification.

### 3.6. Silane Coupling Agent-Modification of Other Clays

Other clays such as vermiculite, talc, serpentine, etc., are also often modified with silane coupling agents in order to obtain better flame retardant properties.

Vermiculite is formed by hydrothermal alteration of clay minerals such as black mica and chrysoberyl or weathering of black mica [92]. Its structural formula is (Mg,Fe,Al)_8_(Si,Al)_4_O_10_(OH)_2_·4H_2_O [93], belonging to the 2:1 type of clay minerals. Vermiculite has a high cation exchange capacity. Its cation exchange capacity is up to about 180 mmol/100g^−1^ [94]. Because of this characteristic, there is less literature on organic modification of vermiculite by silane coupling agents. Song et al. [95] prepared rigid polyurethane/expanded vermiculite composites using KH-550-treated expanded vermiculite as fillers. The LOI of the composites increased from 18.3% to 21.1%, when the filling amount of expanded vermiculite at 3–6 mm particle size reached 30%. This increase in LOI of the composites is due to the fact that expanded vermiculite is noncombustible, so the addition of expanded vermiculite improves the flame retardancy of the composites. Lyu et al. [96] combined a flame retardant (ammonium polyphosphate) and KH-550-modified expanded vermiculite to prevent waste polyurethane from combustion. The LOI of the composites can be up to 30.27%. KH-550-modified expanded vermiculite can exist in an inorganic/organic exfoliation state in polyurethane. The layer of expanded vermiculite distributed in the polymer sheets has good gas–liquid barrier properties. Therefore, the expanded vermiculite layer on the burning surface can block the migration of small combustible molecules to the burning interface which are generated by the decomposition of polyurethane molecular chains. At the same time, it can also slow down the rate of migration of oxygen to the combustion interface. Therefore, the flame retardant system of the ammonium polyphosphate and expanded vermiculite is to some extent conducive to improving the flame retardant property of the material.

Talc is a 2:1 type of clay, and its chemical formula is 3MgO·4SiO_2_·H_2_O. Yang et al. [97] researched the effect of the addition of KH-550-modified talc on the flame retardant properties of polyamide 6/melamine cyanurate (PA6/MCA). When the talc content was 25 phr., the LOI of the composite reached 28.5%, and the UL-94 rating reached V-0. With the addition of talc, the flame retardant properties of the composites were significantly improved in comparison to the PA6/MCA system. The flame retardant effect of MCA on PA6 is mainly manifested in two aspects. On the one hand, it takes away the heat through the molten droplets, and on the other hand, it generates inert gas to dilute the oxygen in the air. However, it cannot enable the formation of a continuous, dense protective layer. The talc modified by KH-550 is uniformly coated on the surface of the material, and it can enhance the cohesive phase flame retardant effect of the composite materials. In summary, the above flame retardant system effectively inhibits combustion under the combined effect of gas phase and condensed phase.

Serpentine is a 1:1-type trioctahedral clay with the chemical formula Mg_3_(Si_2_O_5_)(OH)_4_. Fiber serpentine, also known as chrysotile asbestos, has excellent tensile strength and thermal stability, and it also has low thermal conductivity, good thermal insulation, high electrical resistivity, and high insulating properties. T. Seckin et al. [98] modified chrysotile asbestos using KH-550 and synthesized a polyamidoacetic acid solution from pyromellitic acid dianhydride and 4,4′-diaminodiphenyl ether. Then polyimide–chrysotile composites were prepared with the polyamidoacetic acid solution and the modified chrysotile asbestos. When the mass fraction of the chrysotile asbestos was increased to 5%, the peak exothermic rate of the composite was reduced by 65% compared to that of pure polyimide. This may be due to the strong interaction between chrysotile and polymer, which restricts the movement of polymer chains.

## 4. Clay Grafted with Flame Retardant by Silane Coupling Agent

Flame retardant molecules are able to achieve functionalized modification of clays by grafting them with silane coupling agents.

**Figure 14:**
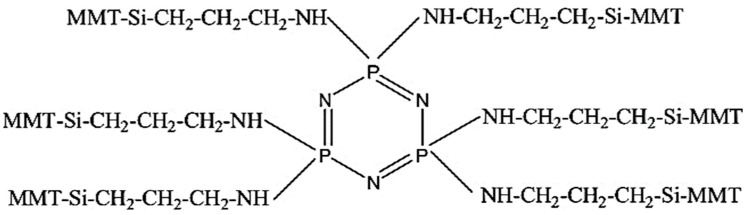
Schematic structure of the new flame retardant [99].

A new flame retardant can be formed by grafting MMT with the phosphorus flame retardant hexachlorocyclotriphosphazene through silane coupling agent. This flame retardant has excellent flame retardant properties. Wang et al. [99] prepared a novel flame retardant by chemically bonding MMT with hexachlorocyclotriphosphonitrile using KH-550. The structure of the flame retardant is shown in Figure 14. Then, the flame retardant was added to polyethylene terephthalate (PET) to study its flame retardant properties. The novel flame retardant improved the LOI of PET from 24.0% to 31.5%, and the materials achieved a UL-94 V-0 rating. The excellent flame retardant properties can be attributed to the synergistic flame retardant effects of phosphorus and nitrogen and the unique properties of MMT. Phosphorus and nitrogen act as flame retardants to dilute the concentration of combustible gases generated during combustion and reduce the exothermic heat of the composite material. MMT acts as a heat insulator. They together improve the flame retardancy of the materials.

Bao et al. [100] synthesized intumescent flame retardant functionalized MMT (IFR-MMT) using diphenyl chlorophosphate, KH-540, etc., as organic modifiers of MMT. And they used this flame retardant on ethylene propylene diene monomer composites. When the mass fraction of the IFR-MMT added was 25%, the composites achieved a UL-94 rating of V-0, and the LOI was 31.8%. When MMT was grafted with intumescent flame retardant through a silane coupling agent, the synergistic effect of the intumescent flame retardant and MMT strengthened the intumescent carbon layer and improved the flame retardancy of the polymer matrix composites.

Chen et al. [101] used KH-550 to graft fullerene C60 with MMT to prepare C60-modified MMT hybrids (C60-Si-MMT). Then, PP and C60-Si-MMT composites were prepared by melting. They found that C60-Si-MMT uniformly dispersed in the matrix after MMT and C60 were chemically grafted by silane coupling agent. The layered structure of MMT blocked the gas release during polymer degradation, and fullerene C60 can capture the highly reactive free radicals generated during heating, thereby inhibiting the oxidative degradation of polymers. A combination of the two through a silane coupling agent can result in a better perform in their respective roles.

**Figure 15:**
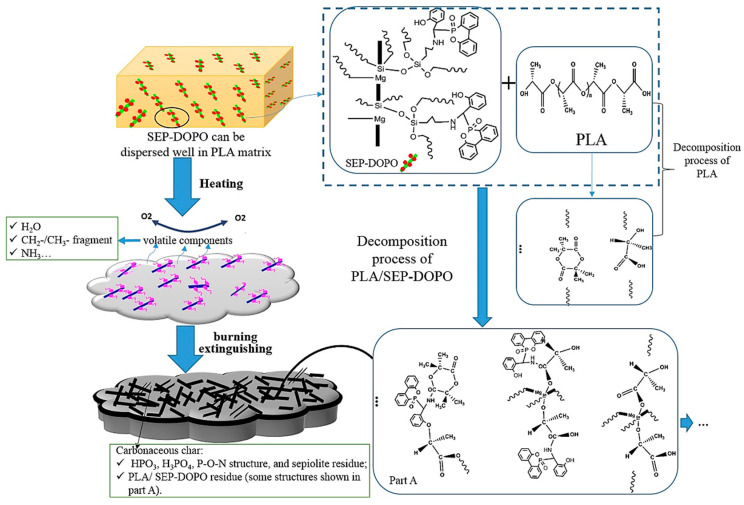
Flame retardant mechanism of PLA/SEP-DOPO composites [103].

9,10-Dihydro-9-oxa-10-phosphaphenanthrene-10-oxide (DOPO) is one of the most popular additive flame retardants [102]. It can form a new flame retardant (SEP-DOPO) by grafting a silane coupling agent with SEP. Jiang et al. [103] grafted DOPO on the surface of KH-550-modified SEP, and then introduced SEP-DOPO into polylactic acid (PLA). The SEP-DOPO improved the flame retardancy and thermal stability of the PLA nanocomposites, and the effect was better than the simple addition of SEP and DOPO. The flame retardant mechanism is shown in Figure 15. When the content of SEP-DOPO was 10%, the LOI of the composites increased from 20.2% to 31.5%, and the UL-94 test achieved V-0. The reactive hydroxyl reaction between SEP-DOPO and the polymer during decomposition promotes the generation of residual carbon in the condensed phase. In addition, the phosphorus-containing groups, ammonia and H_2_O produced by the decomposition of SEP-DOPO are also free radical scavengers that capture free radicals in the gas phase during the combustion process.

DOPO can also be grafted with Kaol through a silane coupling agent. Peng et al. [104] grafted DOPO with Kaol by KH-560 to obtain a novel flame retardant. They found that the addition of 1.0% this flame retardant could significantly enhance the flame retardant properties of polypropylene composites. The LOI of the composites increased from 18% to a maximum value of 28.1%, and the time at which the first molten drop occurred was extended to 54 s. This new flame retardant not only improves the height of the char expansion but also makes the char more continuous and denser. Such residual carbon effectively slows down the flow of heat and combustible gases between the substrate and the atmosphere and cuts off the source of substrate combustion.

**Figure 16:**
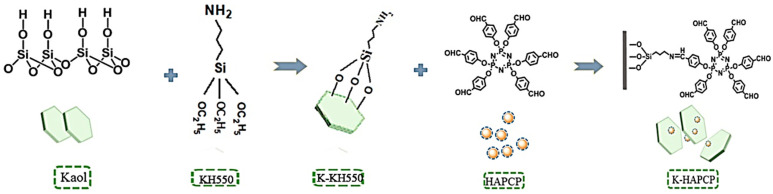
The synthetic routes of K-HAPCP [105].

Hexakis (4-aldehyde phenoxy) cyclotriphosphazene (HAPCP) is a nonhalogenated and highly efficient flame retardant. It can be grafted with Kaol by silane coupling agent. Zhang et al. [105] grafted HAPCP with Kaol using KH-550 to obtain a novel flame retardant (K-HAPCP). The synthetic route is shown in Figure 16. They added it with IFR to polybutylene succinate. After testing, the samples containing 22 wt% IFR and 3 wt% K-HAPCP achieved an LOI of 40.3% and a UL-94 V-0 rating. This is due to the fact that the new flame retardant formed a stable and dense carbon layer that effectively inhibited the release of smoke and prevented the escape of incomplete combustion products.

Phytic acid (PA), is a natural organic phosphate compound extracted from plant seeds. Zhang et al. [106] obtained a novel flame retardant (PA-g-Kaol) by grafting PA with Kaol via APTES, and studied the synergistic flame retardant effect of PA-g-Kaol on EVA/IFR composites. The LOI of EVA was 20.9%; with the addition of 20 wt% IFR, the LOI increased to 24.9%; after replacing IFR with 2 wt% Kaol, the LOI increased to 28.6%; and when replacing IFR with 2 wt% PA-g-Kaol, the LOI of the composites reached 30.8%, with a significant reduction in dripping. The PA-g-Kaol produced inert gases during combustion, which diluted the concentration of flammable gases. The PA-g-Kaol also promoted the formation of a dense char layer, thus improving the flame retardant properties of the composites.

The synergistic flame retardant mechanism of clay nanofillers is attributed to the fact that the nanofillers promote the formation of thicker and reinforced carbon layers in the condensed phase, which effectively prevents heat transfer and mass loss. Composites containing only flame retardants tend to show discontinuous residual char with cracks after combustion, and heat is more likely to penetrate these char layers, resulting in poorer fire performance. However, composites containing clay nanofillers and flame retardants tend to form more continuous and denser char layers, which effectively inhibit heat and mass transfer between the flame and the matrix. Grafting the flame retardant with the clay via silane coupling agent and then filling it into the polymer resulted in essentially the same dispersion position of the flame retardant and the clay in the polymer matrix. The synergistic flame retardant effect of the two is mostly reflected in the condensed phase. The same dispersion position in the matrix makes the residual char of the composite materials more homogeneous and denser during combustion, and enhances the barrier effect to heat and combustible gases. In conclusion, the use of silane coupling agents to chemically graft flame retardant molecules onto clay is more advantageous than the simple addition of flame retardants and clay to a polymer matrix. The advantage of this method is that the method not only improves the dispersion of the clay in the polymer matrix but also more fully utilizes the synergistic effect of the clay and the flame retardant. However, due to factors such as production costs, facilities and equipment, and production processes, this type of flame retardant functionalization modification can only be studied in the laboratory, and large-scale production in factories is currently unable.

## 5. Current Challenges and Potential Future Trends

Despite advancements, challenges such as optimizing the modification process and fully understanding the interactions between modified clay and polymers remain. Future research should focus on these aspects to further enhance the effectiveness of flame retardant polymer composites. The main aspects that need to be improved include the following:

(1) In order to give clay a better dispersion effect in the matrix and to enhance the compatibility of clay and organic polymer materials more effectively, it is necessary to develop silane coupling agents with better modification effects for different matrices. The key to developing silane coupling agents is to change the organic functional groups of silane coupling agents through organic synthesis reaction and other methods, so that the silane coupling agents can achieve a more efficient combination with a single-component specific organic matrices, or can be developed for multi-component organic matrices. Regardless of dry modification or wet modification, further optimization of the modification process is needed. It is recommended to study the optimal modification conditions such as modification temperature and modification time for different silane coupling agents and different clays, and it is also recommended to study the effect of the composite modification of silane coupling agents and other modifiers, as well as to optimize the ratio of the modifiers.
(2) Research on silane coupling agent-modified clay for flame retardant polymers has focused on MMT, SEP, ATP, and Kaol, but few researchers have conducted comparative studies on the flame retardant effects of different silane coupling agent-modified clays in the same polymer matrix. A comparative study of the effects of different clays modified by silane coupling agents and the flame retardant effect of different clays in a polymer matrix after modification can be a future research direction.
(3) While improving the flame retardant properties of clay composites by modifying the clay surface with silane coupling agents, it is also necessary to consider the effects on other properties of the composites, such as mechanical properties, water and corrosion resistance, processability, and degradability. The development of new silane coupling agents and the incorporation of specific functionalized components in composites are feasible solutions for this purpose.
(4) Most silane coupling agent-modified clays for flame retardant composites can still only be realized in the laboratory. In order to meet the requirements of industrialized production, there is still a need for breakthroughs in production costs, facilities and equipment, and production processes. Especially for dry modification, which is less researched at present, it is recommended to explore low-cost and high-efficiency modifiers and modification environments, as well as more concise and efficient operational processes for modification under the premise of meeting the standards of industrial applications.

## 6. Conclusions

In this work, an overview of silane coupling agent-modified clays has been presented first. In this section, silane coupling agents have been categorized according to their organic functional groups. The mechanism of silane coupling agent-modified clay has been presented. Chemical bonding theory and surface infiltration theory have been discussed prominently. The process of silane coupling agent-modified clay has been categorized into wet modification and dry modification. These two modification processes have been then described and their advantages and disadvantages have been compared. The advantages of the wet modification process are that there are many adjustable factors in the process, better dispersion of modifier, uniform surface coating, etc., so the modification efficiency of wet modification is higher. The disadvantage of wet modification is that compared with dry modification, the wet modification process requires pre-slurry configuration, postfiltration, drying, dehydration, and other aspects of the process, making it complex and increasing the modification cost. On the contrary, although the efficiency of dry modification is lower than that of wet modification, its process is simple and the cost is low. For these reasons, wet modification is often used in laboratory research, and dry modification is usually applied in industrial production. This has resulted in the wet process being relatively mature, and there are abundant studies on the related modification mechanisms. However, there is little exploration of the dry process. A large number of laboratory research results have neglected the transition to industrial production. In order to make silane coupling agent-modified clays more widely and efficiently used in the industrial production of flame retardant composites, researchers should do more work on dry modification, such as revealing the mechanism of dry modification and proposing the factors affecting its efficiency.

In another part of this work, silane coupling agent-modified clays for hindering polymer combustion have been presented. The mechanisms of silane coupling agent-modified clays in hindering polymer combustion were summarized into the following three aspects: charcoal-forming barrier mechanism, free radical trapping mechanism, and flame retardant mechanism of cross-linked silane molecules. Then, past research results on silane coupling agent-modified clays (MMT, SEP, ATP, Kaol, etc.) used to prevent polymer combustion were discussed. Nano-clay has the advantages of large specific surface area, good dispersibility, small additive amount, strong interfacial structure effect, etc., which are not possessed by other inorganic fillers. Therefore, it has an immeasurable space and prospect for the development of flame retardant polymer materials. Nano-clay can also produce surprising synergistic flame retardant effects with some flame retardants. However, unmodified nano-clay is often difficult to disperse uniformly in polymers. The use of silane coupling agents provides a way to solve this problem. Silane coupling agents, which are commonly used as modifiers of nano-clay, bridge the gap between polymers and nano-clay. Silane coupling agents have been shown to improve the dispersion of nano-clay in the polymer matrix, effectively reducing the occurrence of agglomeration. Therefore, silane coupling agent-modified clays added to polymers can improve the flame retardant properties of polymers.

It is worth mentioning that the silane coupling agent can also graft flame retardant molecules onto the clay. The flame retardant functionalization of the clay is thus achieved. This more fully utilizes the synergistic flame retardant effect between the clay and the flame retardant. Despite advancements, challenges such as optimizing the modification process and fully understanding the interactions between modified clay and polymers remain. Therefore, at the end of this work, current challenges and potential future trends are presented.

## Figures and Tables

**Table 1:** Common silane coupling agents.

Categorization	Name	Nickname	Chemical Formula
Vinyl	Trichlorovinylsilane	WD-26, A-150, VTCS	C_2_H_3_Cl_3_Si
	Triethoxyvinylsilane	WD-20, A-151, VTEO	C_8_H_18_O_3_Si
	Vinyltrimethoxysilane	WD-21, A-171, VTMO	C_5_H_12_O_3_Si
	Vinyl tris(2-methoxyethoxy) silane	WD-27, A-172	C_11_H_24_O_6_Si
	Vinyltriacetoxysilane		C_8_H_12_O_6_Si
Chlorocarbon	3-Chloropropyltrichlorosilane		C_3_H_6_Cl_4_Si
	3-Chloropropyl(trimethoxy)silane	WD-31, A-143	C_6_H_15_ClO_3_Si
	3-Chloropropyltriethoxysilane	WD-30	C_9_H_21_ClO_3_Si
Aminohydrocarbon	(3-Aminopropyl)triethoxysilane	KH-550, APTES, WD-50	C_9_H_23_NO_3_Si
	3-(Trimethoxysilyl)-1-propanamine	KH-540, WD-56	C_6_H_17_NO_3_Si
	N-[3-(Trimethoxysilyl)propyl]ethylenediamine	KH-791, WD-51	C_8_H_22_N_2_O_3_Si
Epoxy hydrocarbon	3-Glycidoxypropyltrimethoxysilane	KH-560, WD-60, A-187	C_9_H_20_O_5_Si
	Triethoxy(3-glycidyloxypropyl)silane	WD-62	C_12_H_26_O_5_Si
	3-Glycidyloxypropyl(dimethoxy)methylsilane	WD-61	C_9_H_20_O_4_Si
	2-(3,4-Epoxycyclohexyl)ethyltriethoxysilane	A-186	C_14_H_28_O_4_Si
Methacryloxyalkyl	3-(Trichlorosilyl)propyl methacrylate		C_7_H_11_Cl_3_O_2_Si
	3-Methacryloxypropyltrimethoxysilane	KH-570, WD-70, A-174	C_10_H_20_O_5_Si
	3-Methacryloxypropylmethyldimethoxysilane	KH-571, WD-71	C_10_H_20_O_4_Si
Sulfur-containing hydrocarbons	Trimethoxysilylpropanethiol	KH-590, WD-80, A-189	C_6_H_16_O_3_SSi
	3-Mercaptopropyltriethoxysilane	KH-580, WD-81	C_9_H_22_O_3_SSi
	Bis[3-(Triethoxysilyl)propyl]tetrasulfide	WD-40,Si-69	C_18_H_42_O_6_S_4_Si_2_
	Bis(triethoxysilylpropyl)disulfide	WD-42,Si-75	C_18_H_42_O_6_S_2_Si_2_

## Data Availability

Data availability is not applicable to this article as no new data were created or analyzed in this study.

## Supplementary Materials

The following supporting information can be downloaded at: https://www.mdpi.com/article/10.3390/molecules29174143/s1, Table S1: Effect of silane coupling agent-modified clay on epoxy resin.
