# Supplementary material for: Progress in Application of Silane Coupling Agent for Clay Modification to Flame Retardant Polymer
Source: Molecules. 2024 Aug 31;29(17):4143. doi: 10.3390/molecules29174143 (PMC11397132; doi:10.3390/molecules29174143)
Supplement: Supplementary file 1 [file molecules-29-04143-s001.zip › molecules-3167882-supplementary.pdf]

Table S1 Effect of silane coupling agent-modified clay on epoxy resin.

Clay	Silane coupling agent	Other modifiers	Synergistic flame retardant	Optimal additive quantity (%)	LOI (%)	UL-94	Peak heat release rate (kW • m^{-2})	Total heat release (MJ • m^{-2})	Total smoke rate (m^2 • m^{-2})	Glass transition temperature (℃)	Tensile strength (MPa)	Elongation at break (%)	Flexural strength (MPa)	Izod notched impact strength (kJ • m^{-2})	Elastic modulus (GPa)	referen ce
MMT	KH-550			1.5						165	66.00				2.28	[56]
MMT	KH-550/ KH-560/ KH-602	cetyltrimet hylammon ium bromide		3.0						94.4/ 91.3/ 95.2	66.14/ 58.50/ 61.04	3.61/ 3.01/ 3.06	100.78/ 99.27/ 96.33	16.49/ 14.35/ 10.23		[58]
SEP	KH-570			7.0	22.0	V-2	876.0									[66]
SEP	KH-570	aluminium phosphate		20.0	30.1	V-0	1065.1	196.9	4556.5		52.60	7.9	69.6	6.6		[68]
SEP	KH-550		nano-SiO_2	6.0	30.3	V-0	860.9	130.1			76.99	3.46		1.12	3.53	[70]
Kaol	KH-550	TiO_2, SiO_2		3.0	27.3		602.2	81.0	3087.1							[89]
